# Multicenter Evaluation of an Edge-to-Edge Repair System in High-Risk Patients With Degenerative Mitral Regurgitation

**DOI:** 10.1016/j.shj.2026.100793

**Published:** 2026-01-06

**Authors:** Kai Xu, Junjie Zhang, Da Zhu, Yan Wang, Hong Jiang, Jing Chen, Jiyan Chen, Jiancheng Xiu, Ben He, RuiYan Zhang, Xiaogang Guo, ChuanYu Gao, ZhiXiong Zhong, Ping Li, DaJun Chai, Jun Jin, Yuguo Chen, Lang Hong, Yida Tang, Guosheng Fu, Ling Tao, Yining Yang, Shenghua Zhou, Bin Liu, Lijun Gan, Bo Yu, Maurizio Taramasso, Xiangbin Pan, Shaoliang Chen, Yaling Han

**Affiliations:** a State Key Laboratory of Frigid Zone Cardiovascular Disease, Cardiovascular Research Institute and Department of Cardiology, General Hospital of Northern Theater Command, Shenyang, People's Republic of China; b Department of Cardiology, Nanjing First Hospital, Nanjing Medical University, Nanjing, Jiangsu, China; c Department of Structural Heart Disease, Fuwai Yunnan Cardiovascular Hospital, Kunming, Yunnan, China; d Department of Cardiology, Xiamen Cardiovascular Hospital Xiamen University, Xiamen, Fujian, China; e Department of Cardiology, Renmin Hospital of Wuhan University, Wuhan, Hubei, China; f Department of Cardiology, Guangdong Provincial People's Hospital, Guangzhou, Guangdong, China; g Department of Cardiology, Nanfang Hospital of Southern Medical University, Guangzhou, Guangdong, China; h Department of Cardiology, Shanghai Chest Hospital, Shanghai, China; i Department of Cardiology, Ruijin Hospital Affiliated to Shanghai Jiao Tong University School of Medicine, Shanghai, China; j Department of Cardiology, The First Affiliated Hospital, Zhejiang University School of Medicine, Hangzhou, Zhejiang, China; k Department of Cardiology, Fuwai Central China Cardiovascular Hospital, Zhengzhou, Henan, China; l Department of Cardiology, Meizhou People's Hospital, Meizhou, Guangdong, China; m Department of Cardiology, Yulin First People's Hospital, Yulin, Guangxi, China; n Department of Cardiovascular, The First Affiliated Hospital of Fujian Medical University, Fuzhou, Fujian, China; o Department of Cardiology, The Second Affiliated Hospital of the Army Medical University, ChongQing, China; p Department of Emergency Medicine, Qilu Hospital of Shandong University, Jinan, Shandong, China; q Department of Cardiology, Jiangxi Provincial People's Hospital, Nanchang, JiangXi, China; r Department of Cardiology and Institute of Vascular Medicine, Peking University Third Hospital, Beijing, China; s Department of Cardiology, Sir Run Run Shaw Hospital, Zhejiang University School of Medicine, Hangzhou, Zhejiang, China; t Department of Cardiology, The First Affiliated Hospital of Air Force Medical University, Xian, Shaanxi, China; u Department of Cardiology, People’s Hospital of Xinjiang Uygur Autonomous Region, Urumqi, Xinjiang Uygur Autonomous Region, China; v Department of Cardiovascular Medicine, The Second Xiangya Hospital of Central South University, Changsha, Hunan, China; w Department of Cardiology, The Second Hospital of Jilin University, Changchun, Jilin, China; x Department of Cardiology, Affiliated Hospital of Jining Medical University, Jining, Shandong, China; y Department of Cardiology, The Second Affiliated Hospital of Harbin Medical University, Harbin, Heilongjiang, China; z Department of Cardiac Surgery, Herzzentrum Hirslanden Zürich, Zürich, Switzerland

**Keywords:** Degenerative mitral regurgitation, Transcatheter edge-to-edge repair, Transcatheter mitral valve repair

## Abstract

**Background:**

Transcatheter edge-to-edge repair (TEER) has become an effective alternative for treating degenerative mitral regurgitation (DMR) in patients at high surgical risk. The SQ-Kyrin-M TEER system (SQ-Kyrin-M system) is a novel TEER device developed in China. This study aimed to evaluate the feasibility, safety, and 12-month clinical efficacy of the SQ-Kyrin-M system in patients with high-risk degenerative mitral regurgitation.

**Methods:**

In this prospective, multicenter, single-arm study (ClinicalTrials.gov: NCT06467110), 120 patients with symptomatic DMR (grade ≥3+) had the device implanted. The primary endpoint was the clinical success rate at 12 months. Secondary endpoints included technical, device, and procedural success rate; New York Heart Association (NYHA) class improvement; Kansas City Cardiomyopathy Questionnaire (KCCQ) score change; and mitral regurgitation (MR) reduction. Safety endpoints encompassed all-cause mortality, cardiovascular mortality, and major adverse event rate.

**Results:**

A total of 120 patients received the TEER procedure across 25 participating sites in China; the mean age was 71.9 years, and the mean Society of Thoracic Surgeons (STS) risk score was 9.3. At 12 months, the Kaplan–Meier estimates were 82.5% for clinical success, 7.6% for all-cause mortality, and 10.8% for major adverse events; MR ≤2+ and MR ≤1+ were achieved in 91.7 and 70.4% of the patients, respectively; 88.9% of patients were in NYHA class I or II; and KCCQ score had improved by 18.9 points. Favorable left ventricular remodeling was observed with sustained reductions in left ventricular end-diastolic and end-systolic volumes.

**Conclusions:**

This study demonstrates that the SQ-Kyrin-M system is a safe and effective therapeutic option for DMR patients.

## Highlights

• Demonstrates the safety and efficacy of a novel TEER device in high-risk DMR.
• Achieves durable mitral regurgitation reduction and symptomatic improvement.
• Supports the expansion of effective TEER treatment options worldwide.

## Graphical abstract

The SQ-Kyrin-M® transcatheter edge-to-edge mitral valve repair system with 12-month safety and performance outcomes. The device sizing table illustrates the parameters of four clip sizes to accommodate varying anatomical structures. The Kaplan–Meier curve presents the 12-month clinical success rate with 95% CIs, demonstrating superiority over the historical performance goal. The stacked bar chart shows the distribution of MR severity at baseline to 12-month follow-up in the implant population. All subjects presented with grade 3+ or 4+ at baseline, and 91.7% of subjects achieved MR reduction to grade ≤2+ at 12 months. Device-related complications through 12 months included five SLDA events, two leaflet damage events, and five TEER reinterventions. CI, confidence interval; TTE, transthoracic echocardiography; MR, mitral regurgitation; MVR, mitral valve repair; NL, narrow long; NS, narrow short; SLDA, single leaflet device attachment; WL, wide long; WS, wide short.

**Figure:**
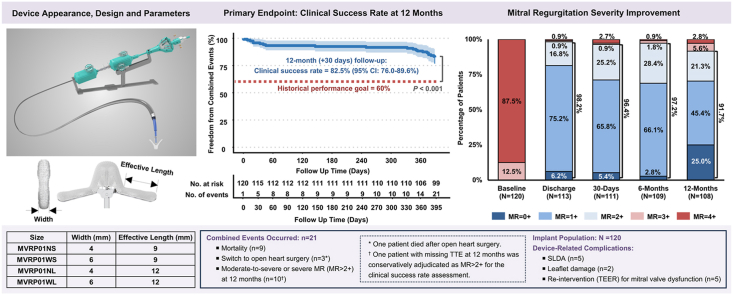

## Introduction

Mitral regurgitation (MR) is among the most prevalent valvular heart diseases in China; it affects approximately 7.5 million individuals who require clinical intervention.^1^ Of these, around 50% are caused by degenerative mitral regurgitation (DMR).^2^^,^^3^ DMR primarily results from degenerative changes in the mitral valve, such as prolapse or rupture of the chordae tendineae, often accompanied by flail-like lesions.^4^ Transcatheter edge-to-edge repair (TEER) of the mitral valve has become a guideline-recommended,^5^ safe, and effective treatment option for high-risk DMR patients due to its minimally invasive nature and expedited recovery profile.^6^^,^^7^ The SQ-Kyrin-MTEER system (SQ-Kyrin-M system), a domestically developed TEER device in China, is conceptually similar to the MitraClip but has distinguishing and unique features. Building on the demonstrated safety and efficacy of the SQ-Kyrin-M system from early first-in-human and functional MR trials,^8^^,^^9^ this study aims to further evaluate the safety and performance of the SQ-Kyrin-M system in patients with DMR.

## Methods

### Trial Design and Patient Selection

The SQ-Kyrin-M DMR trial was a prospective, multicenter, single-arm study. It enrolled clinically symptomatic patients with chronic moderate-to-severe (3+) or severe (4+) DMR deemed high surgical risk or unsuitable for conventional surgery by a multidisciplinary team. High surgical risk was defined as a Society of Thoracic Surgeons score of ≥8 for valve replacement, ≥6 for valve repair, or the presence of two or more indicators of frailty as judged by the cardiac team according to the Valve Academic Research Consortium-2 criteria.^10^

Eligible participants were ≥18 years old and in New York Heart Association (NYHA) functional class II, III, or ambulatory IV. Echocardiographic inclusion criteria were defined following the Endovascular Valve Edge-to-Edge REpair Study (EVEREST)^11^ criteria and comprised left ventricular end-systolic diameter ≤60 mm, primary regurgitant jet originating predominantly within the A2/P2 scallops, flail width ≤15 mm, flail gap ≤10 mm, leaflet length >10 mm, and mitral valve orifice area ≥4.0 cm^2^. Patients with generally accepted contraindications for TEER were excluded from this study.^12^ Anatomical suitability was assessed independently by a designated eligibility committee. Exclusion criteria included previous mitral valve surgery; infective endocarditis; untreated severe coronary artery disease; recent acute myocardial infarction, transient ischemic attack, or stroke within 30 days; pulmonary hypertension (>70 mmHg); life expectancy <12 months; severe tricuspid regurgitation; left ventricular ejection fraction (LVEF) <20%; and calcified landing zones. The complete inclusion and exclusion criteria are shown in Supplementary Table 1. Follow-up visits (including echocardiography and other assessments) occurred immediately postprocedure; before discharge; at 1, 6, and 12 months; and annually up to 5 years. All echocardiograms were analyzed by an independent core laboratory (Supplementary Table 2), and all major adverse events (MAEs) were adjudicated by an independent clinical events committee.

All implantations were performed according to the standardized implantation procedure, which was conducted under general anesthesia with endotracheal intubation. Transoesophageal echocardiography and fluoroscopic guidance were performed in a hybrid operating room/catheterization laboratory.

### Ethical Conduct

The study protocol was approved by local ethics committees at each site, and all patients provided written informed consent. The trial was conducted in accordance with Good Clinical Practice principles, ISO 14155:2020, and the Declaration of Helsinki. The trial is registered at ClinicalTrials.gov (NCT06467110).

### The SQ-KYRIN-M System

The SQ-Kyrin-M system comprises a 24F delivery guiding catheter and a clip delivery system. While conceptually similar to the MitraClip G4 system, including the independent grasping design and availability in four clip sizes, the SQ-Kyrin-M offers a more cost-effective treatment option for patients with TEER indication. Furthermore, it introduces multiple modifications to enhance procedural safety: a larger leaflet contact area that distributes stress and reduces the risk of leaflet rupture, an optimized cabling design to reduce the risk of chordae entanglement, and a three-layer locking mechanism in the clip delivery system that ensures sufficient locking and provides better steerable stability and accuracy.

### Trial Endpoints

The primary endpoint was the clinical success rate at 12 months, which was a composite endpoint defined as freedom from all-cause death, surgery for mitral valve dysfunction, or residual MR >2+. The performance goal was set at 60% based on prior studies including EVEREST II,^13^ EVEREST High-Risk Registry,^14^ and ACCESS EU Phase I.^15^

Secondary endpoints included technical success, device success, procedural success rate, reduction in MR, and functional status improvements assessed by NYHA class and Kansas City Cardiomyopathy Questionnaire (KCCQ) score. Technical success was defined as successful deployment and leaflet grasping with retrieval of the delivery system without death or emergency surgery related to the device or procedure. Device success was achieved when MR was ≤2+ immediately postprocedure, and procedural success entailed maintaining device success without MAEs at 30 days. MAEs included death, stroke, myocardial infarction, or cardiovascular surgery due to device- or procedure-related adverse events occurring after transfemoral access and atrial septal puncture. Safety endpoints encompassed all-cause mortality, cardiovascular death, and MAE rate at 1, 6, and 12 months.

### Echocardiographic Assessments

Both transthoracic echocardiography and transesophageal echocardiography were performed at local sites following acquisition protocols provided by the independent core laboratory primarily for patient screening and procedure planning. Follow-up transthoracic echocardiography evaluations were analyzed by the core laboratory in accordance with the American Society of Echocardiography guidelines.^16^^,^^17^

### Statistical Analysis

This study was designed to demonstrate superiority over a historical performance goal of 60% for the primary endpoint. A sample size of 118 evaluable patients was estimated to provide 80% power to detect a significant increase in this study. Normal distribution of reported variables was tested with the Shapiro-Wilk test. Continuous variables are reported as mean ± standard deviation (SD) or median (interquartile range) as appropriate. Categorical variables are presented as frequencies and percentages. For comparisons across time points, paired *t*-tests were used for continuous variables, while Wilcoxon signed-rank tests or McNemar tests were applied for categorical variables. Time-to-event data were analyzed using the Kaplan–Meier or competing risks method. No adjustments for multiple comparisons were made. All tests were two-sided with a significance level of 0.05. Analyses were performed using SAS version 9.4 (SAS Institute, Cary, North Carolina).

## Results

### Patient Population and Baseline Characteristics

Between March 2022 and November 2023, 123 patients were enrolled at 25 participating sites in China (Supplementary Table 3). The modified intent-to-treat population included 120 patients who received the investigational device. The flowchart is presented in Supplementary Figure 1. The mean age was 71.9 ± 5.2 years, and 44.2% (53/120) were female. The prevalent comorbidities included coronary heart disease (50.0%), peripheral vascular disease (44.2%), chronic obstructive pulmonary disease (20.8%), prior stroke or transient ischemic attack (15.8%), and previous cardiac valve surgery (2.5%). Of the 120 patients, 73.3% (88) were in NYHA functional class III or higher (Table 1). The cohort's mean STS risk score was 9.3 ± 1.4 (for mitral valve replacement), indicating high risk for conventional surgical intervention. All patients exhibited MR grade ≥3+ with 87.5% (105/120) having MR grade ≥4+ (Table 2).

**Table 1:** Baseline characteristics

Baseline characteristics	mITT (N = 120)
Age, y	71.9 ± 5.2 (120)
Female sex	44.2 (53/120)
STS score for mitral valve replacement∗	9.3 ± 1.4 (115)
STS score for mitral valve repair∗	7.0 ± 0.6 (4)
Hypertension	64.2 (77/120)
Diabetes	16.7 (20/120)
Smoke	34.2 (41/120)
Prior hospitalization for heart failure within 1 y	70.8 (85/120)
NYHA class	
II	26.7 (32/120)
III	59.2 (71/120)
IVa	14.2 (17/120)
Previous PCI	20.0 (24/120)
Previous stroke or TIA	15.8 (19/120)
Peripheral vascular diseases	44.2 (53/120)
Pulmonary hypertension	64.2 (77/120)
Cerebrovascular disease	21.7 (26/120)
Chronic kidney disease	20.8 (25/120)
Coronary heart disease	50.0 (60/120)
Prior cardiac valve surgery	2.5 (3/120)
History of major surgery/injury	22.5 (27/120)
Chronic obstructive pulmonary disease	20.8 (25/120)
KCCQ-OS score, point	68 [52, 77] (119)

*Notes.* Values are mean ± SD (n), median [Q1, Q3] (n) or % (n/N).

Abbreviations: KCCQ-OS, Kansas City Cardiomyopathy Questionnaire Overall Summary; mITT, modified intent-to-treat; NYHA, New York Heart Association; PCI, percutaneous coronary intervention; STS, Society of Thoracic Surgeons; TIA, transient ischemic attack.

∗ As most of the published articles report the STS score as mean ± SD, here we also show it as mean ± SD to be comparable.

**Table 2:** Echocardiographic variables at baseline

Echocardiographic measures	mITT (N = 120)
MR severity, %	
3+	12.5 (15/120)
4+	87.5 (105/120)
Effective regurgitation orifice area, cm^2^	0.54 [0.41, 0.71] (111)
LVESD, mm	36.0 ± 6.5 (119)
LVEDD, mm	54.0 ± 6.8 (120)
LVESV, mL	47 [36, 63] (118)
LVEDV, mL	128 [104, 160] (120)
LAV, mL	95 [79, 125] (67)
LVEF, %	63 [60, 67] (119)
Prolapse width, mm	11.0 [8.9, 14.0] (120)
Prolapse/flail gap, mm	5.6 [4.0, 7.0] (120)
Length of anterior, mm	24.4 ± 4.6 (120)
Length of posterior, mm	17.0 [13.6, 19.0] (120)
Calcification of mitral valve leaflets, %	1.7 (2/120)

*Notes.* Values are mean ± SD (n), median [Q1, Q3] (n) or % (n/N).

Abbreviations: LAV, left atrial volume; LVEDD, left ventricular end-diastolic diameter; LVEDV, left ventricular end-diastolic volume; LVEF, left ventricular ejection fraction; LVESD, left ventricular end-systolic diameter; LVESV, left ventricular end-systolic volume; mITT, modified intent-to-treat; MR, mitral regurgitation.

### Procedural Outcomes

Successful implantation was achieved in 120 patients (97.6%). Of the three unsuccessful implantations, there were two cases of unsuitable anatomy on intraoperative transesophageal echocardiography and one case of pericardial effusion during transseptal puncture. The median device implantation time was 74 minutes, and the median total procedural duration was 101 minutes. On average, 1.5 ± 0.6 clips were used per patient. Specifically, 55.8% (67/120) of patients received one clip, 41.7% (50/120) received two clips, and 2.5% (3/120) received three clips. Additional procedural details are summarized in Table 3.

**Table 3:** Procedural characteristics

	mITT (N = 120)
Technical success, %	100 (120)
Device implantation time, min∗	74 [47, 104] (120)
Procedural time, min†	101 [74, 137] (120)
Fluoroscopy time, min	46 [26, 69] (109)
Number of devices implanted per patient, %	
1	55.8 (67)
2	41.7 (50)
3	2.5 (3)
Average number of devices implanted	1.5 ± 0.6 (120)
Size of device, %	
MVRP01NL	10.8 (19)
MVRP01NS	13.6 (24)
MVRP01WL	68.8 (121)
MVRP01WS	6.8 (12)

*Notes.* Values are mean ± SD (n), median [Q1, Q3] (n) or % (n).

Abbreviations: mITT, modified intent-to-treat; MVR, mitral valve repair; NL, narrow long; NS, narrow short; WL, wide long; WS, wide short.

∗ Device implantation duration: from when the guide sheath reaches the left atrium to when the device delivery system returns to the guide sheath.

† Procedural duration: from transseptal start to guide sheath removal from the left atrium.

### Primary Endpoint

Throughout the entire year of follow-up, there were no instances of loss to follow-up. The Kaplan–Meier estimate of the clinical success rate at 12 months was 82.5%; nine patients died, three patients underwent surgery for mitral valve dysfunction, and ten patients had MR >2+ (Graphical Abstract). Of the three surgical cases, one was due to single leaflet device attachment (SLDA), while the other two were due to persistent and worsening regurgitation.

### Secondary Endpoints

#### Efficacy Endpoints

**Figure 1:**
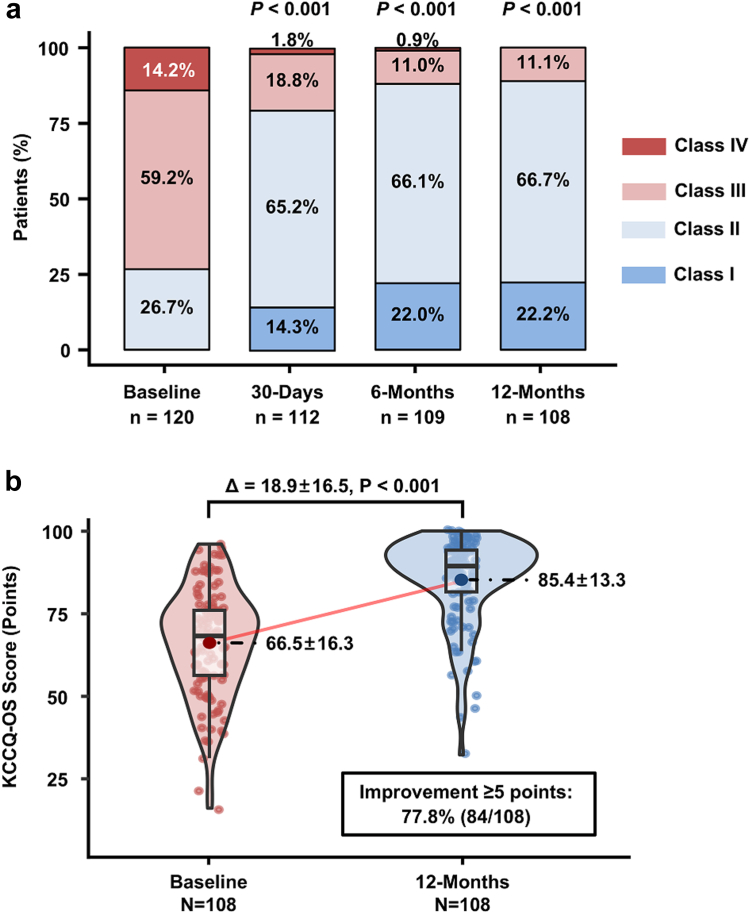
Functional status improvement in patients treated with the SQ-Kyrin-M system. Ordinal variables are presented as frequencies and continuous variables are expressed as mean ± SDs. (a) Change in the New York Heart Association (NYHA) class for symptoms of heart failure. The graph shows unpaired data, and the *p* values were calculated using the Wilcoxon signed-rank test for paired patients compared with baseline data. (b) Kansas City Cardiomyopathy Questionnaire Overall Summary (KCCQ-OS) score from baseline to 12 months. 108 patients with available data for both time points are included in the paired analysis, and *p* value is calculated based on the paired samples *t*-test.

Among patients who received the investigational device, the immediate postprocedure technical success rate was 100% (120/120). Device success rate at 30 days was 93.8% (106/113), and procedural success at 30 days was 92.0% (104/113). Patients exhibited significant symptomatic improvement, with 79.5% (89/112) in NYHA classes I/II at 30 days, increasing to 88.1% (96/109) at 6 months and 88.9% (96/108) at 12 months. The KCCQ overall summary score improved significantly from baseline to 12 months, with an average increase of 18.9 ± 16.5 points (*p* < 0.001, Figure 1).

#### Performance Endpoints

**Figure 2:**
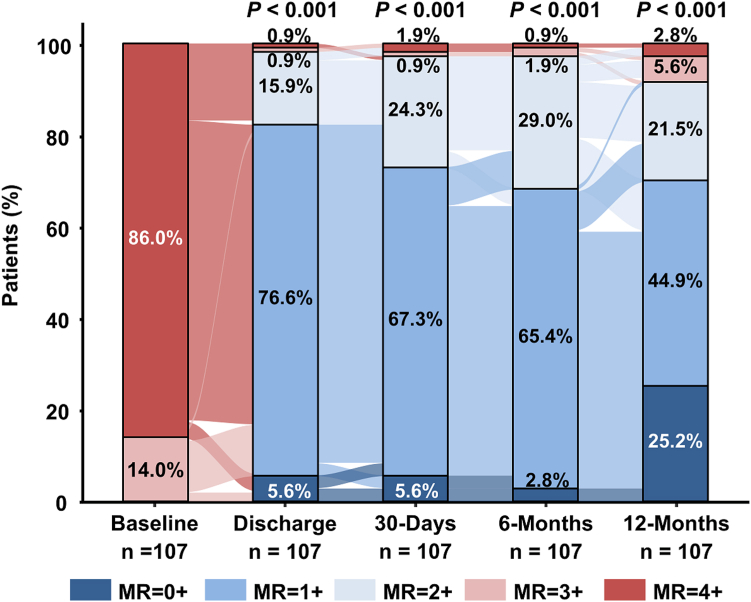
Alluvial diagram of the mitral regurgitation severity. Graph shows paired data. The *p* values were calculated using the Wilcoxon signed-rank test compared with baseline data. MR severity was assessed by an independent echo core laboratory (ECL) using transthoracic echocardiography. Abbreviation: MR, mitral regurgitation.

The proportion of patients with MR ≤2+ was 98.2% at discharge, 96.4% at 30 days, 97.2% at 6 months, and 91.7% at 12 months. Statistically significant improvements from baseline were observed at all time points (*p* < 0.001) as illustrated in the alluvial diagram (Figure 2). MR ≤1+ was observed in 71.2% at 30 days, 68.8% at 6 months, and 70.4% at 12 months with sustained reduction over time (Supplementary Figure 2).

**Figure 3:**
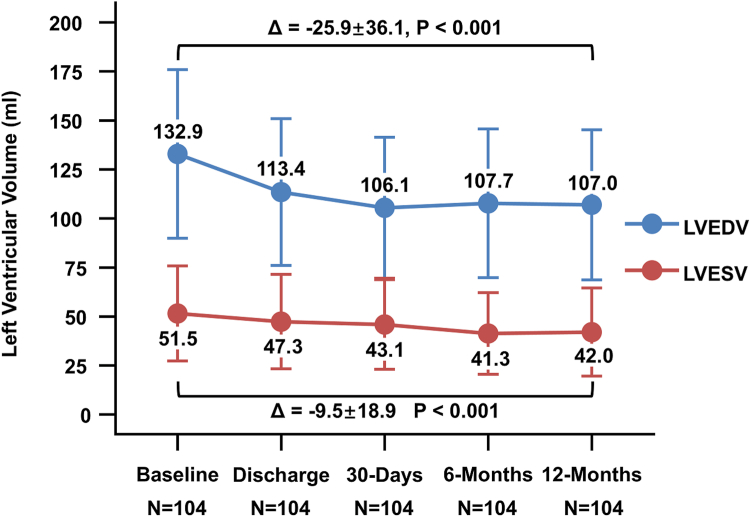
Change in left ventricular end-diastolic/systolic volume. One hundred and four patients with available data for all time points are included in the paired analysis. Means are reported for each time point and error bars represent SDs. The change of left ventricular volume from baseline to 12 months is represented by Δ, and *p* values are calculated based on the paired samples *t*-test. Abbreviations: LVEDV, left ventricular end-diastolic volume; LVESV, left ventricular end-systolic volume.

Left ventricular remodeling was also evident: left ventricular end-diastolic volume decreased from 132.9 ± 42.9 mL at baseline to 107.0 ± 38.3 mL at 12 months (*p* < 0.001), and left ventricular end-systolic volume decreased from 51.5 ± 24.3 mL to 42.0 ± 22.5 mL (*p* < 0.001, Figure 3). Although average LVEF decreased at discharge (60.1%) compared to baseline (62.8%), it nearly recovered to 62.8% at 12 months (*p* = 0.91, Supplementary Figure 3). The transmitral mean pressure gradient increased from 3.05 ± 1.65 mmHg at baseline to 3.78 ± 2.13 mmHg at 12 months (mean difference 0.72 mmHg, *p* = 0.005).

#### Safety Endpoints

The Kaplan–Meier estimated rate of MAEs at 12 months was 10.8% (Table 4). These included nine all-cause deaths (7.6%) (Supplementary Figure 4), three postoperative strokes (2.5%), and three surgeries for mitral valve dysfunction (2.5%). The entire burden of surgical conversion was observed within the first 60 days postprocedure (Supplementary Figure 5). All deaths were due to cardiovascular causes as adjudicated by a clinical events committee. No myocardial infarction occurred.

**Table 4:** Estimated cumulative incidence of major adverse events and device-related complications at 12 months

No. of events (%)	mITT (n = 120)	95% CI (%)
MAE	13 (10.8)	5.1-16.2
All-cause death	9 (7.6)	2.7-12.2
Myocardial infarction	0 (0.0)	-
Stroke	3 (2.5)	0.7-6.7
Surgery for mitral valve dysfunction	3 (2.5)	0.7-6.6
Device-related complications	9 (7.5)	3.7-13.1
Single-leaflet device attachment	5 (4.2)	1.6-8.9
Leaflet damage	2 (1.7)	0.3-5.4
Reintervention for mitral valve dysfunction	5 (4.2)	1.6-8.9

*Notes.* The cumulative incidence and the 95% CI of safety events were analyzed using Kaplan–Meier or competing risks method.

Abbreviations: CI, confidence interval; MAE, major adverse event; mITT, modified intent-to-treat.

Under the competing risks analysis, the cumulative incidence of device-related complication was 7.5%, including five cases of SLDA (4.2%), two of leaflet damage (1.7%), and five of TEER reintervention (4.2%). The five SLDA events occurred under the following circumstances: one was associated with mitral annular calcification and ultimately required conversion to surgery; another case, in which Barlow's morphology was not fully recognized initially, was diagnosed during reintervention; a third resulted from echocardiographic artifact caused by the first clip, which compromised stable positioning of the second clip; the fourth was managed conservatively after detection; and the final case was complicated by suspected infective endocarditis, and the patient ultimately died.

## Discussion

This study demonstrates that the SQ-Kyrin-M system is a safe and effective treatment option for high-risk patients with DMR. The trial successfully met its prespecified primary efficacy endpoint. The device achieved substantial and durable MR reduction accompanied by favorable left ventricular reverse remodeling, improved NYHA functional class, and enhanced quality of life. These findings support the safety, efficacy, and clinical utility of this innovative Chinese transcatheter edge-to-edge mitral repair device for managing DMR.

The SQ-Kyrin-M system exhibited a favorable safety profile with a low incidence of MAEs. The estimated all-cause mortality at 12 months in this study was 7.5%. This result is lower than those reported in earlier registries from the previous decade—including the EVEREST II High-Risk Registry, the Real World Expanded Multicenter Study of the MitraClip System (REALISM) study (22.8%),^18^ the Transcatheter Valve Treatment Sentinel Pilot Registry (TVCT) (15.3%),^19^ and the transcatheter mitral valve interventions (TRAMI) registry (19.8%)^20^—and remains comparable to outcomes from key contemporary studies published in 2023, such as the MitraClip EXPAND Study of the Next Generation of MitraClip Devices (EXPAND) (12.5%)^21^ and EXPAND G4 (8.4%)^22^ registries in their DMR populations.

The incidence of SLDA in this study was 4.2%, which is slightly higher than the rates in the Edwards PASCAL Transcatheter Valve Repair System Pivotal Clinical Trial (CLASP IID) (2.0%)^23^ and EXPAND G4 (1.6%). These cases reflect a combination of anatomical complexity, early learning experience, and procedural challenges during the initial adoption of the device. A learning-curve analysis comparing the first three cases per center with later cases demonstrated comparable clinical success (*p* = 0.28) but a significantly higher rate of MR >2+ at 12 months in the initial group (14.8 vs. 3.0%, *p* < 0.05) (Supplementary Figure 6).

A key strength of the SQ-Kyrin-M device lies in its durable MR reduction, with 98.2% of patients achieving MR ≤2+ at discharge and 91.7% maintaining this at 12 months. By comparison, MR ≤2+ was achieved in 81.6% of patients in the EVEREST II trial and 93.8% in the EXPAND. Emerging evidence^24^^,^^25^ indicates that residual MR ≤1+ is associated with improved long-term outcomes. In this cohort, 81.4% of patients had MR ≤1+ at discharge, and 70.4% maintained this level at 12 months; these findings highlight the device’s effectiveness and long-term durability. To assess the impact of residual MR severity, patients with MR ≤2+ at 12 months were stratified into those achieving optimal reduction (MR ≤ 1+) versus lesser reduction (MR = 2+). Although the differences in left ventricular volume reduction (i.e., left ventricular end-diastolic volume and left ventricular end-systolic volume) between subgroups were not statistically significant, a consistent numerical trend toward greater reverse remodeling was observed in the MR ≤1+ group. This suggests that striving for MR ≤1+ may promote more substantial reverse remodeling, highlighting the potential long-term value of optimizing procedural results (Supplementary Table 4). Univariate and multivariate logistic regression analyses indicated that lower baseline MR grade, absence of hypertension, and a smaller flail gap were associated with achieving MR ≤1+ at 12 months (Supplementary Table 5). An early decline in LVEF likely reflected an acute hemodynamic response following MR correction. LVEF subsequently recovered to near-baseline levels by 12 months and was accompanied by sustained reductions in left ventricular volumes. These findings mirror those reported in the EXPAND and G4 studies, thus reinforcing the potential of TEER to promote beneficial reverse remodeling over time.

The device provided substantial symptomatic and functional improvement. At 12 months, the proportion of patients in NYHA class I/II and the improvement in KCCQ-Overall Summary score were comparable to the results reported in contemporary registries such as EXPAND and EXPAND G4. Importantly, 77.8% of patients achieved a clinically meaningful improvement (≥5-point increase in KCCQ score),^26^ further underscoring the durable symptom relief associated with the SQ-Kyrin-M system.

The combination of high technical success, effective MR reduction, symptomatic benefits, and low adverse event rates suggests that the SQ-Kyrin-M device is a promising addition to the TEER landscape. Despite the early stage of TEER implementation in China, a high level of procedural efficiency, including a median procedure time shorter than that in early Western series, was observed. This efficiency is attributable in part to the integration of remote proctoring and an effective learning adaptation by operators. The experience demonstrates that focused training and systematic proctoring can facilitate the rapid attainment of procedural proficiency in new markets while offering a replicable model to expedite TEER adoption and skill dissemination worldwide. Contributing to accumulating clinical evidence on a global scale, long-term follow-up of the present cohort is planned through 5 years to further evaluate the durability of the treatment. Furthermore, the development and ongoing evaluation of the SQ-Kyrin-M system represent a meaningful step toward enhanced international collaboration in structural heart disease, and they showcase China’s growing capacity for innovation and its potential role in the worldwide effort to advance TEER therapy.

### Limitations

This single-arm study has inherent limitations; the absence of a randomized control group introduces potential selection bias and restricts the direct interpretability of the findings, while comparisons with external historical cohorts are confounded by differences in demographics, anatomical selection, operator experience, and center-specific practices. The relatively short 12-month follow-up limits the assessment of long-term durability, and this study was conducted during the coronavirus disease 2019 (COVID-19) pandemic. In addition, the mean cohort age of 71 years—lower than in many contemporary TEER registries—along with the high surgical risk profile and severe baseline MR may limit direct comparison of mortality rates with other studies and reduce generalizability to lower-risk or older populations.

## Conclusions

This inaugural pivotal study demonstrated that the SQ-Kyrin-M system had a favorable safety and efficacy profile for the treatment of symptomatic DMR. The device achieved durable MR reduction and enhanced functional capacity and a low rate of adverse events. These findings position SQ-Kyrin-M as a promising addition to the TEER therapeutic options. Future studies with longer follow-up durations and comparative designs are necessary to confirm its long-term benefits and wider applicability.

## Review Statement

The review of this manuscript was managed by Guest Editor Rodrigo Estevez-Loureiro, MD.

## Ethics Statement

The study protocol was approved by local ethics committees at each site. The trial was conducted in accordance with Good Clinical Practice principles, ISO 14155:2020, and the Declaration of Helsinki.

## Funding

This work was supported by the National Key Project of Research and Development Plan during the 14th Five-Year Plan Period (2022YFC2503400), the Major Science and Technology Special Plan Project of Yunnan Province (202302AA310045), and the Yunnan Expert Workstation under the Yunnan Provincial Project for Scientific and Technological Talents and Platforms (202305AF150069). The study is sponsored by Shanghai Shenqi Medical Technology Co, Ltd (Shenqi Medical).

## Disclosure Statement

All authors report their institutions receiving institutional research grants from Shenqi Medical for the conduct of the study.
