# Supplementary material for: Multicenter Evaluation of an Edge-to-Edge Repair System in High-Risk Patients With Degenerative Mitral Regurgitation
Source: Struct Heart. 2026 Jan 6;10(5):100793. doi: 10.1016/j.shj.2026.100793 (PMC13129433; doi:10.1016/j.shj.2026.100793)

| **SUPPLEMENTARY MATERIALS**   \|  \| **Page** \| \| --- \| --- \| \| **Supplementary Table 1.** Inclusion and exclusion criteria. \| 2-3 \| \| **Supplementary Table 2.** Independent Echocardiography Core Laboratory (ECL) Personnel and Affiliation \| 4 \| \| **Supplementary Table 3.** Number of enrollments in each site. \| 5 \| \| **Supplementary Table 4.** Comparison of change in left ventricular measurements between subgroups with MR ≤1+ and MR =2+ at 12-month follow-up. \| 6 \| \| **Supplementary Table 5.** Univariate and multivariate analysis of influencing factors for MR ≤1+ at 12-month (Logistic regression) \| 7 \| \| **Supplementary Figure 1.** Flowchart illustrating the enrollment and follow-up. \| 8 \| \| **Supplementary Figure 2.** MR reduction to ≤1 at 30 days, 6 months, and 12 months. \| 9 \| \| **Supplementary Figure 3.** LVEF change from baseline to 12 months. \| 10 \| \| **Supplementary Figure 4.** Overall Survival at 12 months. \| 11 \| \| **Supplementary Figure 5.** Freedom from Surgery for Mitral Valve Dysfunction at 12 months. \| 12 \| \| **Supplementary Figure 6.** Impact of Learning Curve on the Primary Endpoint. \| 13 \|   **Supplementary Table 1. Inclusion and exclusion criteria.** |
| --- | --- | --- | --- | --- | --- | --- | --- | --- | --- | --- | --- | --- | --- | --- | --- | --- | --- | --- | --- | --- | --- | --- | --- | --- |
| **Criteria # Inclusion criteria** |
| **Clinical selection criteria**   1. Severe mitral regurgitation (MR) ≥3+ as determined by transthoracic echocardiography (TTE); 2. Society of Thoracic Surgeons (STS) risk score results show that patients at high risk for traditional surgery or patients who cannot tolerate traditional thoracotomy were judged to be refractory to mitral valve surgery due to a ≥8% risk of death from STS surgery for surgical mitral valve replacement, a ≥6% risk of death from STS surgery for surgical mitral valve repair, or any of the following risk factors:   a. Porcelain aorta or active ascending aortic plaque  b. Mediastinum treated with radiotherapy  c. Past mediastinitis  d. Left ventricular ejection fraction (LVEF) <40%  e. Presence of a patent coronary bypass implant  f. Acceptance of two or more cardiothoracic surgeries  g. Liver cirrhosis  h. Other surgical risk factors (e.g., having ≥2 moderate to severe frailty indicators or other surgical high-risk factors as determined by the cardiac team)   1. Degenerated MR; 2. Age ≥18 years regardless of gender; 3. Classification of cardiac function: NYHA class II, III, or ambulatory class IV; 4. Assessment by a multidisciplinary cardiac team as very high risk or unsuitable for routine mitral valve surgery; 5. Understanding of the objective of the trial, willingness to participate and completion of the Informed Consent Form, willingness to accept the relevant examination and clinical follow-up.   **Anatomy selection criteria:**   1. Left ventricular end-systolic diameter (LVESD) ≤60mm 2. Mitral valve disease prolapse lesion area width ≤15mm, prolapse height ≤10mm, effective length of anterior and posterior valve leaflets >10mm 3. Mitral valve effective orifice area (EOA) ≥4.0cm^2^ 4. No obvious calcification of main grasp mitral valve leaflets 5. Patient anatomy allows atrial septum approach |

| **Criteria # Exclusion criteria** |
| --- |
| 1. History of mitral valve surgery; 2. Infective endocarditis or active infection; 3. Complication with severe untreated coronary artery disease; 4. Pulmonary hypertension (pulmonary systolic blood pressure >70mmHg); 5. Transthoracic echocardiographic evidence suggesting moderate-severe to severe right ventricular dysfunction; 6. Left ventricular ejection fraction <20%; 7. Extreme frailty and intolerance of general anesthesia surgery or experience of the state of shock requiring circulatory support; 8. Diagnosis with hypertrophic cardiomyopathy, restrictive cardiomyopathy, constrictive pericarditis; 9. MR due to active rheumatic heart disease or rheumatic etiology; 10. Severe renal insufficiency (estimated glomerular filtration rate [eGFR] ≤25 mL/min) or requirement of chronic renal replacement therapy; 11. Definite coagulation disorders and severe coagulation system diseases; 12. Clear contraindications to the use of anticoagulants; 13. Stroke or transient cerebral ischemic attack within 30 days; 14. Any intracardiac mass, left ventricular thrombus, or atrial thrombus detected on transthoracic echocardiography; 15. Severe tricuspid regurgitation (TR); 16. Other valve disease requiring surgery or interventional therapy; 17. Severe macrovascular disease requiring surgical treatment; 18. Severe symptomatic carotid stenosis (>70% on ultrasonography) or carotid stenting within 30 days; 19. Inappropriate anatomical structures of the heart and valves indicated by imaging examinations; 20. Known allergy to contrast agents and nickel-titanium memory alloy products; 21. Resting systolic blood pressure <90 mmHg or >160 mmHg; 22. Possession of diseases that seriously affect the evaluation of treatment (e.g., patients with severe neurological lesions affecting cognitive ability, patients with malignant tumors, etc.); 23. Life expectancy <12 months; 24. Severe thoracic deformity; 25. Pregnancy, breastfeeding, or intention to become pregnant within the next 12 months. |

**Supplementary Table 2.** Independent Echocardiography Core Laboratory (ECL) Personnel and Affiliation

| **Echocardiographic Core Laboratory** | | |
| --- | --- | --- |
| Wei Hua, MS | Department of Cardiology, Ruijin Hospital, Shanghai Jiao Tong University School of Medicine, Shanghai, China. | Echocardiologist |
| Xiuxiu Su, MS | Department of Cardiology, Ruijin Hospital, Shanghai Jiao Tong University School of Medicine, Shanghai, China. | Echocardiologist |

**Supplementary Table 3. Number of enrollments in each site.**

| **Site No.** | **Site Name** | **[Enroll](https://zhida.zhihu.com/search?content_id=149147420&content_type=Article&match_order=1&q=Enrollment&zhida_source=entity)ed** |
| --- | --- | --- |
| 01 | General Hospital of Northern Theater Command | 17 |
| 02 | Peking University Third Hospital | 1 |
| 03 | Shanghai Chest Hospital | 4 |
| 04 | Ruijin Hospital Affiliated to Shanghai Jiao Tong University School of Medicine | 4 |
| 07 | Sir Run Run Shaw Hospital, Zhejiang University School of Medicine | 1 |
| 08 | The First Affiliated Hospital, Zhejiang University School of Medicine | 4 |
| 09 | Nanjing First Hospital, Nanjing Medical University | 21 |
| 10 | Guangdong Provincial People's Hospital | 6 |
| 11 | Nanfang Hospital of Southern Medical University | 5 |
| 13 | Yulin First People's Hospital | 2 |
| 14 | Xiamen Cardiovascular Hospital Xiamen University | 12 |
| 15 | First Affiliated Hospital of Fujian Medical University | 2 |
| 18 | The Second Affiliated Hospital of the Army Medical University | 2 |
| 19 | Fuwai Yunnan Cardiovascular Hospital | 14 |
| 20 | The First Affiliated Hospital of Air Force Medical University | 1 |
| 22 | People’s Hospital of Xinjiang Uygur Autonomous Region | 1 |
| 24 | Qilu Hospital of Shandong University | 2 |
| 27 | Fuwai Central China Cardiovascular Hospital | 4 |
| 28 | Renmin Hospital of Wuhan University | 10 |
| 30 | The Second Xiangya Hospital of Central South University | 1 |
| 31 | Jiangxi Provincial People's Hospital | 2 |
| 34 | The Second Hospital of Jilin University | 1 |
| 36 | Affiliated Hospital of Jining Medical University | 1 |
| 37 | Meizhou People's Hospital | 4 |
| 39 | The Second Affiliated Hospital of Harbin Medical University | 1 |

**Supplementary Table 4. Comparison of change in left ventricular measurements between subgroups with MR ≤1+ and MR =2+ at 12-month follow-up.**

|  | 12 months  MR ≤ 1+ (n=76) | 12 months  MR = 2+ (n=23) | P value |
| --- | --- | --- | --- |
| Change in LVEDV  from baseline to 12 months, mL | -27.5 ± 36.4 | -18.4 ± 33.7 | 0.273 |
| Change in LVESV  from baseline to 12 months, mL | -10.2 ± 17.7 | -4.8 ± 20.4 | 0.258 |

Values are mean ± SD. P values were calculated based on Welch Two Sample t-test.

LVEDV = left ventricular end-diastolic volume; LVESV = left ventricular end-systolic volume.

**Supplementary Table 5. Univariate and multivariate analysis of influencing factors for MR ≤1+ at 12-month (Logistic regression)**

| **Characteristic** | **Univariable** | | | | | **Multivariable** | | | | |
| --- | --- | --- | --- | --- | --- | --- | --- | --- | --- | --- |
|  | **N** | **Event N** | **OR** | **95% CI** | **p-value** | **N** | **Event N** | **OR** | **95% CI** | **p-value** |
| **Age** |  |  |  |  |  |  |  |  |  |  |
| < 75 | 86 | 57 | 1.55 | 0.69, 3.49 | 0.288 |  |  |  |  |  |
| ≥75 | 34 | 19 | — | — |  |  |  |  |  |  |
| **Sex** |  |  |  |  |  |  |  |  |  |  |
| 1 | 67 | 44 | 1.26 | 0.60, 2.65 | 0.550 |  |  |  |  |  |
| 2 | 53 | 32 | — | — |  |  |  |  |  |  |
| **Baseline MR** |  |  |  |  |  |  |  |  |  |  |
| 3+ | 15 | 13 | 4.33 | 0.93, 20.19 | 0.062 | 15 | 13 | 3.90 | 0.82, 18.58 | 0.088 |
| 4+ | 105 | 63 | — | — |  | 105 | 63 | — | — |  |
| **Hypertension** |  |  |  |  |  |  |  |  |  |  |
| No | 43 | 32 | 2.18 | 0.96, 4.96 | 0.062 | 43 | 32 | 2.66 | 1.10, 6.42 | 0.030 |
| Yes | 77 | 44 | — | — |  | 77 | 44 | — | — |  |
| **Flail gap** | 120 | 76 | 0.84 | 0.71, 1.00 | 0.048 | 120 | 76 | 0.82 | 0.68, 0.98 | 0.030 |
| **Complexity** |  |  |  |  |  |  |  |  |  |  |
| Green Zone | 100 | 64 | 1.19 | 0.44, 3.17 | 0.735 |  |  |  |  |  |
| Yellow Zone | 20 | 12 | — | — |  |  |  |  |  |  |
| **Number of Clips** |  |  |  |  |  |  |  |  |  |  |
| =1 | 67 | 43 | 1.09 | 0.51, 2.29 | 0.829 |  |  |  |  |  |
| >1 | 53 | 33 | — | — |  |  |  |  |  |  |
| Abbreviations: MR = Mitral Regurgitation, CI = Confidence Interval, OR = Odds Ratio | | | | | | | | | | |

**Supplementary Figure 1. Flowchart illustrating the enrollment and follow-up.** The diagram depicts patient inclusion with visit windows of 30 ± 7 days for the 30-day follow-up and up to 12 months + 30 days for the 12-month follow-up. Three patients did not receive the implant. During the follow-up period, five patients died and two underwent mitral valve surgery within the 30-day window. Of the five, one patient died after surgery. Additionally, four patients died and one patient underwent mitral valve surgery during the 12-month follow-up period.


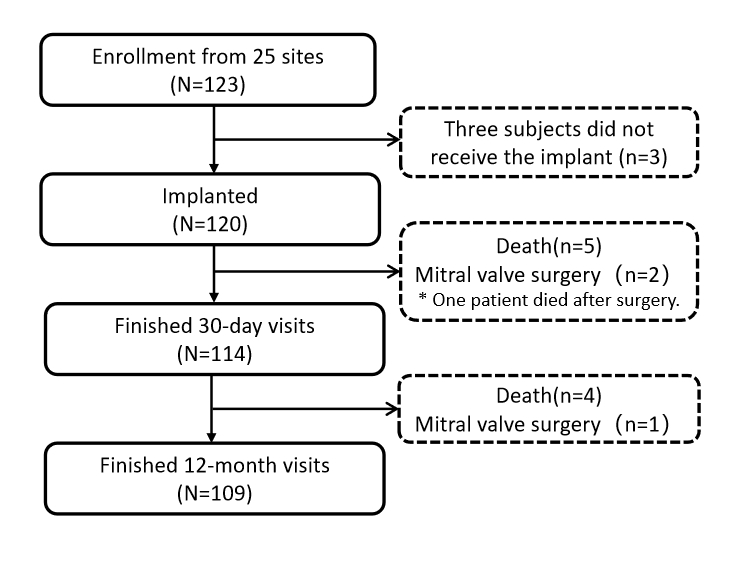


**Supplementary Figure 2. MR reduction to ≤1 at 30 days, 6 months, and 12 months.** Graph shows unpaired data. The p-values were calculated using McNemar's Chi-squared test with continuity correction. MR severity was assessed by the core laboratory using transthoracic echocardiography. MR=mitral regurgitation.


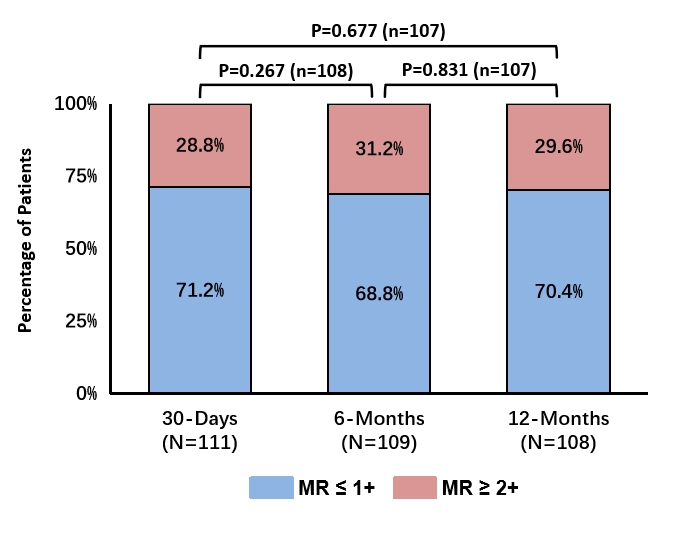


**Supplementary Figure 3. LVEF change from baseline to 12 months.** 104 patients with available data for all time points are included in the paired analysis. Error bars represent mean ± SDs. Δ is the change of LVEF from baseline to 12 months, and p-value is calculated based on the paired samples t-test. LVEF = Left Ventricular Ejection Fractions.


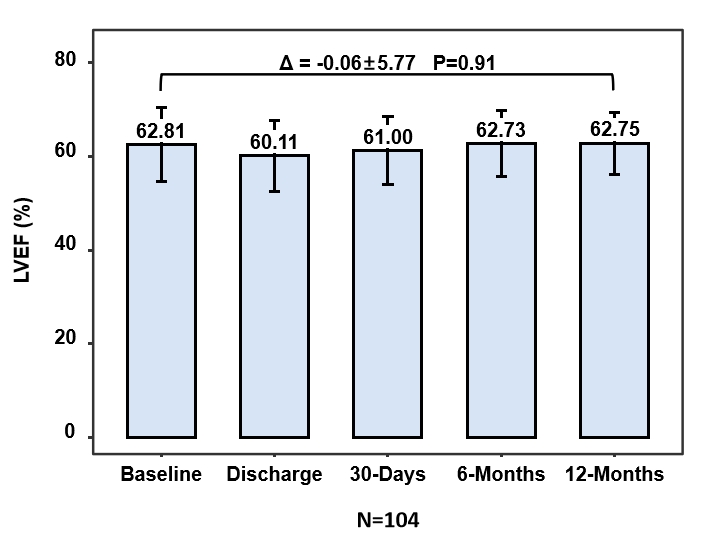


**Supplementary Figure 4. Overall Survival at 12 months.**

Kaplan-Meier analysis shows the overall survival probability for the cohort. The shaded area denotes the 95% confidence interval. The number of patients at risk and number of events over time is presented in the corresponding table.


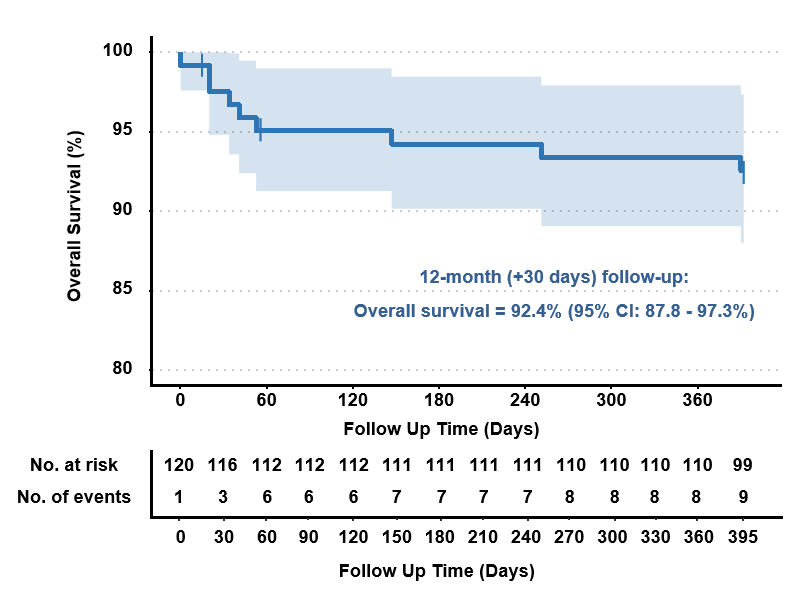


**Supplementary Figure 5. Freedom from Surgery for Mitral Valve Dysfunction at 12 months.**

The curve shows the probability of remaining free from surgical conversion over time in patients initially treated with intervention. This estimate was derived by subtracting the cumulative incidence function (CIF) for surgery from 1 within a competing risks framework, where death was treated as a competing event. The shaded area represents the 95% confidence interval.


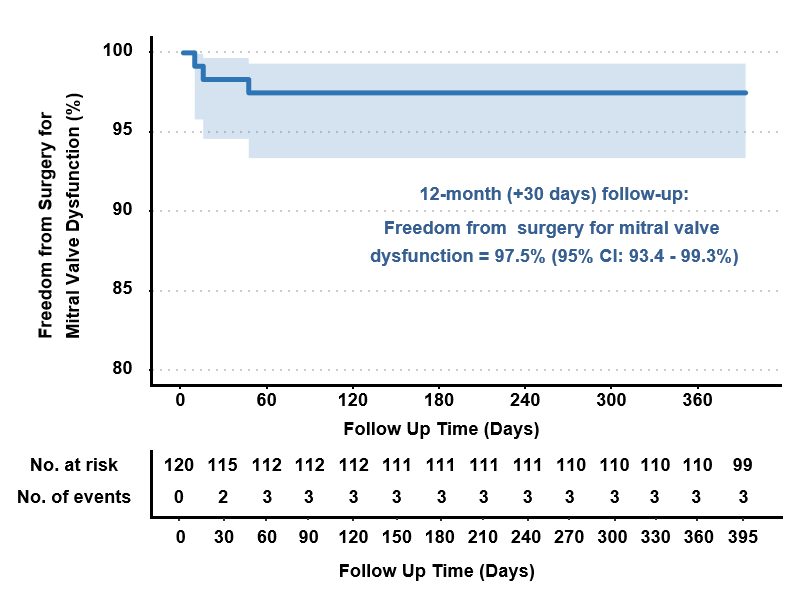


**Supplementary Figure 6. Impact of Learning Curve on the Primary Endpoint.**

Kaplan-Meier curves comparing clinical success rates between initial and subsequent cases. The result suggested that learning curve did not adversely affect early safety outcomes but was associated with a lower efficacy in preventing significant mitral regurgitation at 12 months.


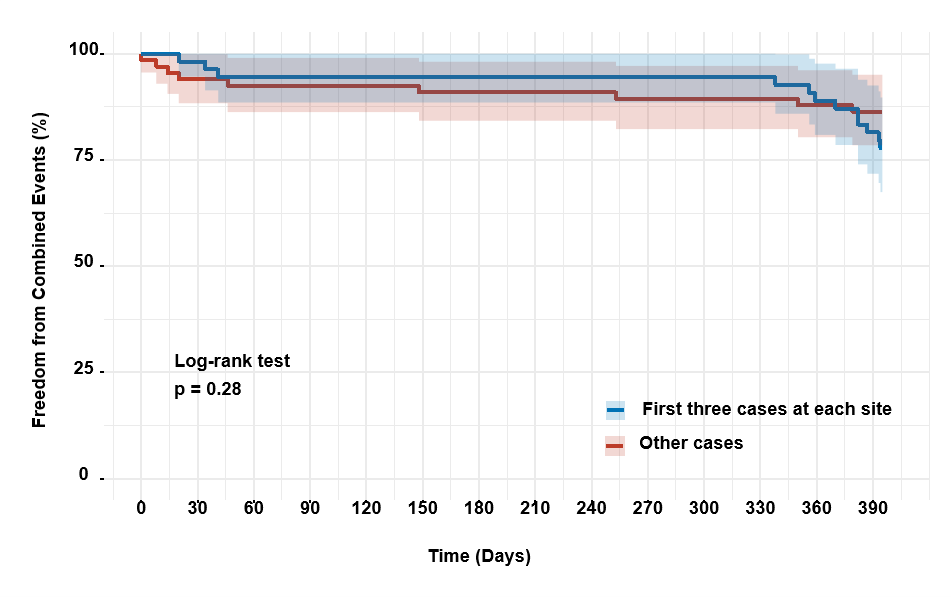

Supplement: Supplementary Materials [file mmc1.docx]
